# Prediction of microbial phenotypes based on comparative genomics

**DOI:** 10.1186/1471-2105-16-S14-S1

**Published:** 2015-10-02

**Authors:** Roman Feldbauer, Frederik Schulz, Matthias Horn, Thomas Rattei

**Affiliations:** 1 Department of Microbiology and Ecosystem Science, Division of Computational Systems Biology, University of Vienna, Althanstr. 14, 1090 Vienna, Austria; 2 Department of Microbiology and Ecosystem Science, Division of Microbial Ecology, University of Vienna, Althanstr. 14, 1090 Vienna, Austria

**Keywords:** Microbes, Prokaryotes, Phenotype, Phenotypic traits, Machine learning, Orthologous groups, Classification, Metagenomics, Intracellular microorganisms

## Abstract

The accessibility of almost complete genome sequences of uncultivable microbial species from metagenomes necessitates computational methods predicting microbial phenotypes solely based on genomic data. Here we investigate how comparative genomics can be utilized for the prediction of microbial phenotypes. The PICA framework facilitates application and comparison of different machine learning techniques for phenotypic trait prediction. We have improved and extended PICA's support vector machine plug-in and suggest its applicability to large-scale genome databases and incomplete genome sequences.

We have demonstrated the stability of the predictive power for phenotypic traits, not perturbed by the rapid growth of genome databases. A new software tool facilitates the in-depth analysis of phenotype models, which associate expected and unexpected protein functions with particular traits. Most of the traits can be reliably predicted in only 60-70% complete genomes. We have established a new phenotypic model that predicts intracellular microorganisms. Thereby we could demonstrate that also independently evolved phenotypic traits, characterized by genome reduction, can be reliably predicted based on comparative genomics.

Our results suggest that the extended PICA framework can be used to automatically annotate phenotypes in near-complete microbial genome sequences, as generated in large numbers in current metagenomics studies.

## Background

Microorganisms, comprising bacteria, archaea and unicellular eukaryotes, are key components of all ecosystems on earth. Their tremendous phylogenetic, ecological and functional diversity is so far only insufficiently understood. Although genome sequencing has within the last two decades enormously advanced the investigation of microbes, microbial genomes have mainly been sequenced from DNA obtained from well-characterized, pure lab cultures. The majority of microbes, however, cannot be cultivated and was therefore inaccessible for genome research [1]. Metagenomic techniques, studying DNA directly obtained from environmental samples, have provided first important insights into genomic features of the unseen majority of microorganisms [recently reviewed in [2]]. Improvements in high-throughput sequencing and DNA extraction protocols, combined with advanced computational methods for binning and taxonomic classification of metagenomic sequences, have recently enabled the reconstruction of near-complete genome sequences of even low-abundant members of microbial communities [3-5]. These recent advances have not only triggered a paradigm shift from "gene-oriented" to "genome-oriented" metagenomics, but also leave us with an emerging bioinformatic problem: the prediction of biological phenotypes and ecological roles of uncharacterized microbial species from their partial genome sequences.

The representation of microbial genomes by their protein-coding genes, associated to orthologous or homologous groups, is the most widely used approach for the organization of large-scale genomic data [6-8]. A wide range of applications, e.g. the prediction of metabolic functions [9] or protein-protein interactions [10], utilize clusters of orthologous groups (COGs). COGs are phylogenetic abstractions of genes on the last universal common ancestor (LUCA) level. NOGs extend this concept to non-supervised groups [7]. So far the highest phylogenetic and ecological diversity of published genome sequences has been achieved for bacteria. During the last years also an increasing number of archaea and unicellular eukaryotes have been included in genome databases [11]. According to fundamental principles of microbial genome evolution, such as the preference for compact and streamlined genomes [12], the presence or absence of COGs in microbial genomes is highly informative. The continuing growth of genome databases therefore holds an enormous potential for advanced computational methods making use of large-scale comparative genomics.

Phenotypic traits of microbes can be very diverse. Structured and computer-readable organization of trait descriptions has been suggested e.g. in the Ontology of Microbial Phenotypes [13]. They range from morphologic and physiological traits to specific molecular or metabolic capabilities. Numerous traits, such as cell envelope structure, as indicated by the Gram stain, have been acquired early in evolution and are therefore encoded in the core section of the pan-genome [14]. Other traits, such as protein secretion capabilities, are evolutionarily more dynamic and are encoded in the variable section of the pan-genome. It can be speculated that the broad evolutionary diversity of microbial traits will be a substantial challenge for computational methods. Generic computational methods, namely those based on large databases of COGs, will most likely represent a first layer of methods for trait prediction. Additional specific models describing well-defined traits based on metabolic and/or regulatory models [e.g. used in [15]] will be needed for a deeper interpretation of a microbial genotype.

Previous work on such generic methods was in many cases limited to searching for one-to-one relations between genes and phenotypic traits. This strategy works very reasonably for simple metabolic traits. E.g., the *amoA *gene encoding ammonia monooxygenase [16] is characteristic for ammonia oxidizing archaea and bacteria. However, single marker genes are of limited predictive power for many other traits. Recent methodological improvements utilize heuristic association rule mining (ARM) to find many-to-one relations. They are based on mutual information [17] or predictive associations [18]. MacDonald and Beiko [18] developed a software framework for comparison of different phenotype prediction methods (PICA), which operates on the level of COG presence or absence in genomes. It includes plug-ins for CPAR and libSVM [19] among others. While PICA features support vector classification with the latter, the original authors focused on ARM and a conditional weighted mutual information metric.

In this study we investigate the application of purely SVM-based classification for phenotypic trait prediction in microbiology and microbial ecology, with an emphasis on metagenomic sequences and vastly increasing data amounts. We further present a novel model for prediction of obligate intracellular microorganisms.

## Methods

### Prediction software and evaluation data sets

Phenotypic trait prediction was performed by machine learning techniques provided by the PICA framework, which is publicly available at http://kiwi.cs.dal.ca/Software/PICA[18]. It provides plug-ins for association rule (CPAR) and support vector classification (SVM) [18]. A mixed approach utilizing association rule mining as a feature-selection step for support vector classification is also available (CPAR2SVM). PICA operates on the level of presence or absence of genetic features, using clusters of orthologous groups by default. It features example data sets based on genotype profiles from the eggNOG 2.0 database [20] and phenotypic trait labels from JGI IMG [21] and NCBI Genomes [11]. We follow the nomenclature used in [18] for ten example traits with slight adaptations: aerobic (aerobe), anaerobic (anaerobe), facultative anaerobic (facult), Gram-negative (gramneg), halophilic (halo), motility (motile), photosynthetic (photo), psychrophilic (psychro), endospore-forming (spore) and thermophilic (therm).

### Genotype data

COG will be used as a collective term for both COGs and NOGs throughout the article. COG profiles were obtained for all genomes from the eggNOG 4.0 database [7], if the corresponding species is either part of its core or periphery set. Otherwise we called genes with PRODIGAL v2.60 using the default translation table [22] and mapped them with the NCBI cognitor software [23] to an in-house generated sequence reference representing all proteins from eggNOG 4.0 COGs.

The training data for intracellular lifestyle comprise 43 obligate intracellular, 6 facultative intracellular and 48 free-living bacterial species. Their taxonomic diversity is depicted in Additional File 1: Figures S1-S3. The data amounts to a total of 97 genomes, containing 30455 unique COGs.

### Phenotypic trait data

The assignment of completely sequenced genomes to obligate intracellular, facultative intracellular, and free-living phenotypes was performed by manual knowledge extraction from scientific literature [such as [24-29]]. All assignments are listed in Additional file 1: Table S1.

### Machine learning and statistics

Association rule mining in PICA is based on the CPAR algorithm [18]. Support vector classification was performed with PICA standard parameters (linear kernel, soft-margin cost parameter C = 5), unless stated otherwise. The feature ranking is based on the absolute values of the linear SVM weights as described in [30]. Prediction qualities are measured as balanced accuracy, i.e. the mean of sensitivity and selectivity as described in Equation (1). They are obtained in 5-fold cross-validation procedures with 10 replicates. True and false predictions are counted per replicate. Balanced accuracies are calculated from these numbers and subsequently averaged over all replicates to obtain mean balanced accuracies and standard deviation.

(1) $$Balanced\;accuracy=\frac{1}{2}\left(\frac{TP}{TP+FN}+\frac{TN}{TN+FP}\right)$$

Legend: TP, TN, FP, FN signify the number of true positive, true negative, false positive and false negative predictions, respectively.

### Scaling the problem size

In order to estimate run time and memory consumption in a scenario of increasing input data sizes, 5000 virtual species were created. They comprise genotype profiles of sizes comparable to average genomes in eggNOG 4.0 [7] with virtual features selected at random from a set of nearly 200,000. This figure was derived from the current number of COGs in eggNOG, and raised slightly as to account for a possible increase in future versions of the database. We expect this number to be higher than absolutely necessary, since not all COGs are relevant to microbes. However, we make use for it as a worst-case scenario and demonstrate feasible phenotype prediction even under unfavorable circumstances. Labels for virtual phenotypic traits were assigned to the virtual genomes at random.

## Results and discussion

### Software development

The PICA framework was initially released in 2010 and has not received further improvements by the original developers since then. We downloaded the open-source code from the project's webpage. The initial release did not highlight SVM as a stand-alone method. Therefore, we re-wrote parts of PICA's SVM plug-in as to enable its use for phenotype prediction in novel genomes. For instance, we ensured that all COGs are always mapped to correct SVM features, especially if not all COGs in the prediction set had already been present in the training data. In addition, we extended the software by implementing a function to extract the most predictive features from linear SVM models. Dumping and ranking features enables the user to interpret the models from a biological perspective: the COGs with the highest discriminative power for presence or absence of a phenotype are ranked at the top. If specific proteins are already known to be part of the genetic underpinning of a phenotype of interest, the user finding the according COGs ranked highly in the list might gain confidence in the biological relevance of the predictions. Unexpected high-ranking COGs hint at previously unknown protein functions associated with the phenotype. However, these may also derive from taxonomic correlations rather than a shared phenotype, which can be caused by insufficient training data. Several minor changes were applied to the software in order to improve code quality. The current release can be obtained from the PICA website or directly from https://github.com/univieCUBE/PICA

### Machine learning considerations

**Figure 1:**
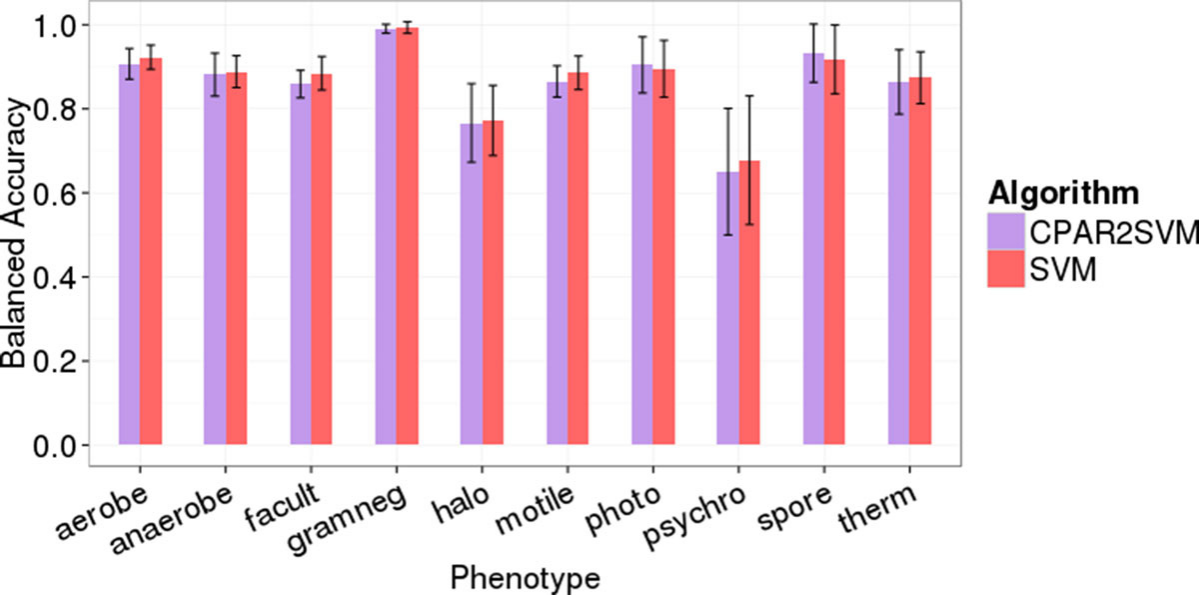
**Phenotype prediction quality of different machine learning techniques**. Quality for ten exemplary traits measured as balanced accuracy in 10 replicate 5-fold cross-validations. Error bars indicate standard deviation.

**Figure 2:**
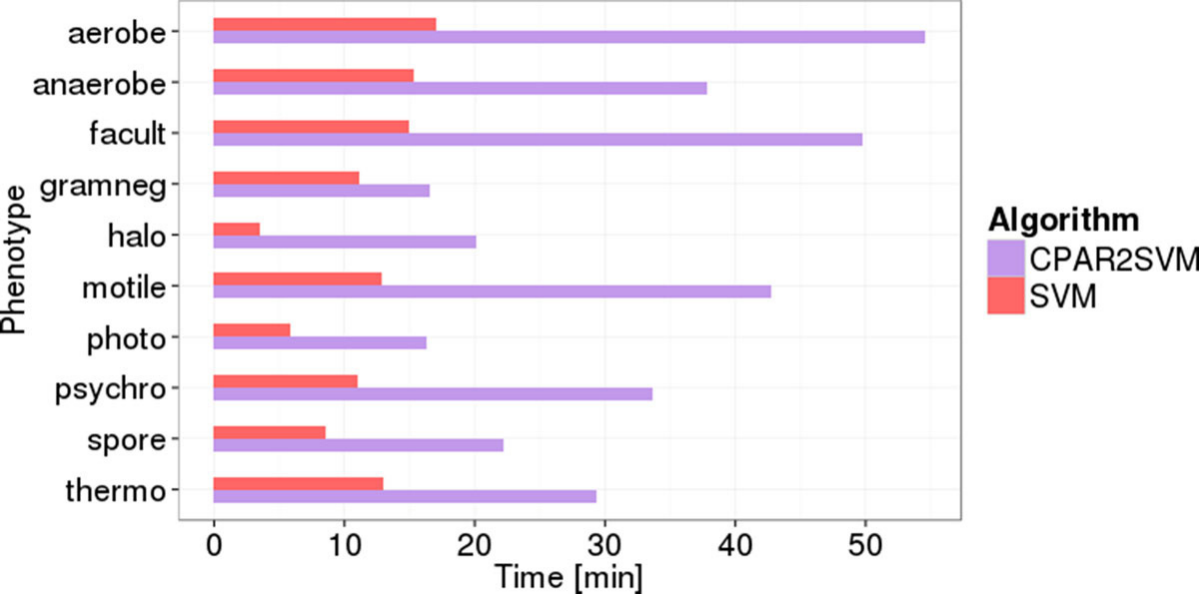
**Effect of different machine learning techniques for phenotype prediction on run time**. Run time for cross-validations described in Fig. 1. This amounts to the combined time for training and testing 50 subsets of the complete data set (plus some overhead).

**Figure 3:**
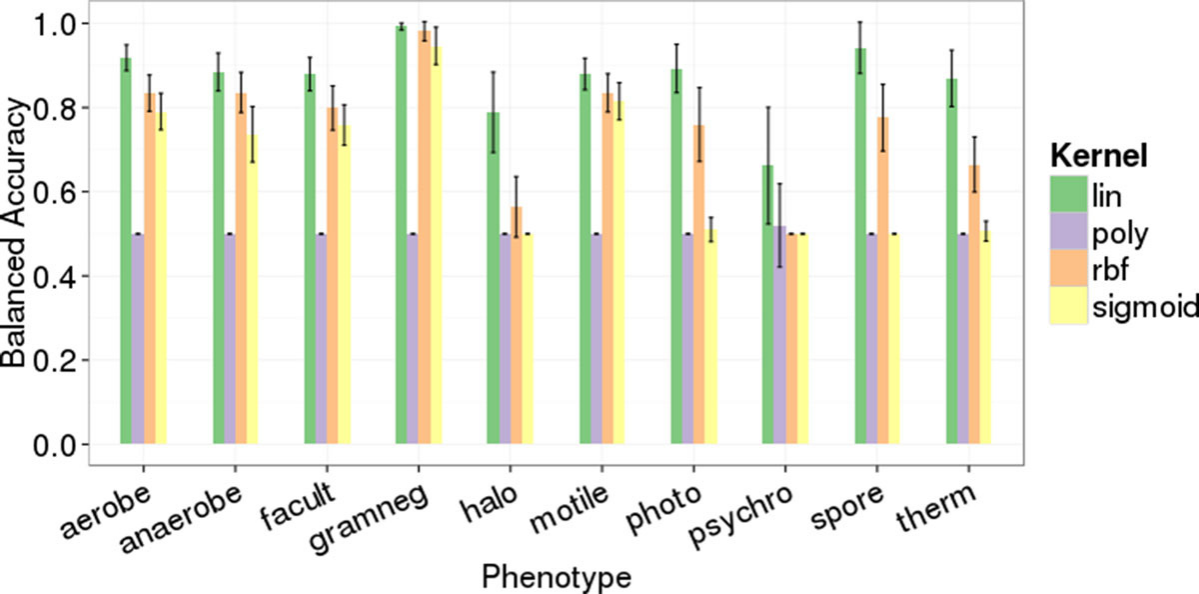
**Phenotype prediction quality for different SVM kernels measured as balanced accuracy in 10 replicate 5-fold cross-validation**. Kernel abbreviations: lin... linear, poly... polynomial, rbf... radial basis function, sigmoid. For each kernel, PICA standard parameters were used. Error bars indicate standard deviation.

The original publication for PICA focused primarily on association rule mining for phenotype prediction [18]. The authors introduced SVMs as an alternative for decision-tree classifiers and trained those only with COGs from association rule antecedents mined beforehand (CPAR2SVM). We re-evaluated the example data sets and performed 10 replicates of 5-fold cross-validation with the SVM and CPAR2SVM approaches. We observed no significant difference in phenotype prediction quality between the two methods (Figure 1). However, the run time was approximately three times lower (average over ten phenotypic traits) using SVM only (Figure 2). Considering these results, we decided to drop association rule mining and performed all further predictions based solely on support vector classification. Furthermore, we repeated the evaluation employing additional SVM kernels. The linear kernel, which is used by PICA by default, is often suboptimal compared to others like e.g. radial basis functions (RBF). Nevertheless, our results clearly show the linear kernel performing better than or at least as good as RBF, sigmoid and polynomial kernels for all ten phenotypic traits (Figure 3). Besides from being most accurate in our test cases, the linear kernel has further general advantages: it is computationally less expensive than other kernels and facilitates feature ranking, which is a non-trivial problem for other kernels.

### Coping with increasing problem sizes

We initially evaluated PICA with its example data set, which comprises COG profiles based on eggNOG 2.0 [20]. This database has been updated to version 4.0 [7] since PICA was released to the public, which brought several changes. One important difference for our purposes is the increasing number of COGs overall as well as specifically on LUCA level. The number the COGs present in at least one of the species from the example data set increased from 47,615 to 192,421. We repeated the evaluation of the ten example phenotypic traits with COG profiles from eggNOG 4.0, while maintaining the original phenotypic trait profiles, in order to estimate the impact of increasing dimensionality on computation time. The average time required for the cross-validation procedure increased from 12 minutes to 43 minutes. Thus, we deem phenotype predictions based on eggNOG 4.0 profiles computationally tractable. No significant differences were observed for prediction accuracy (data not shown).

**Figure 4:**
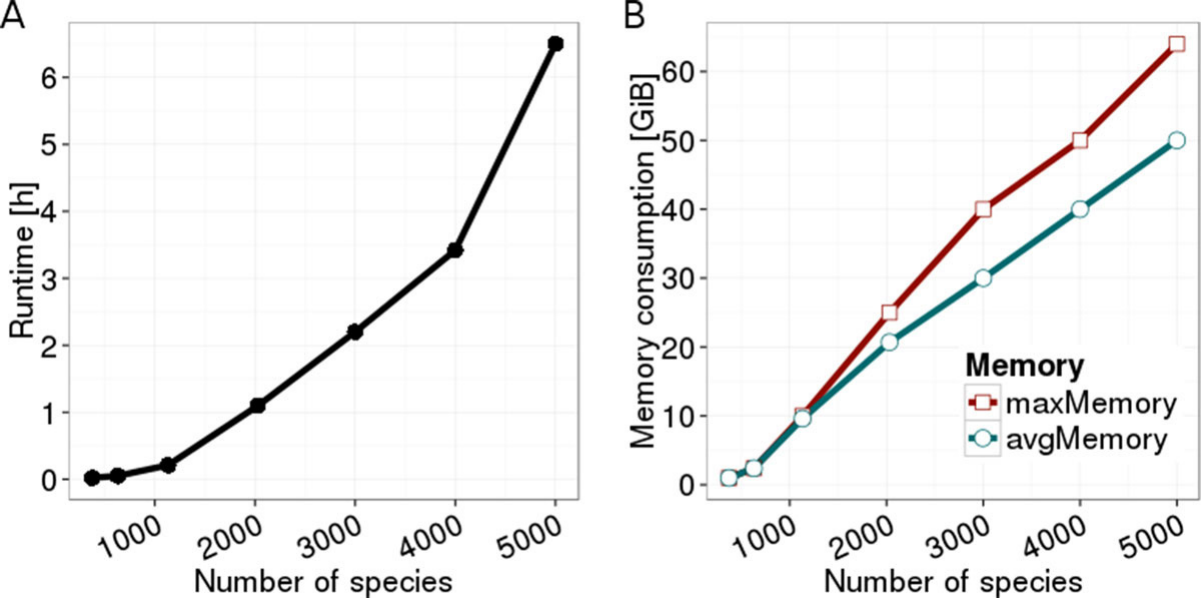
**Computation run time and memory consumption per fold of 5-fold cross-validation of a virtual phenotype for increasing problem sizes**. Virtual species contain average bacteria-sized genomes. Problem dimensionality increases with the number of species to approximately 200,000 for 5000 species. Memory was measured as peak main memory necessary at the beginning of cross-validation (maxMemory) and average memory usage after the peak (avgMemory).

Furthermore, we created virtual species to assess the effect of an increasing number of genomes in training sets in terms of run time and memory consumption. For real traits, it will be impossible to increase the sizes of genome sets to arbitrary numbers, because of insufficient biological knowledge. Several sets of increasing size were compiled and cross-validations were performed for virtual phenotypic traits. Our results indicate that problem sizes of 5,000 genomes with approximately 200,000 different COGs in the training set are still computationally feasible (Figure 4). A typical phenotype prediction scenario currently involves only several hundred genomes with trait labels. We expect the number of COGs not to rise dramatically in upcoming eggNOG versions. Also, not all present COGs are relevant for microbes. Thus, we conclude that PICA is capable of dealing with future large data sets.

### Assessing the applicability for metagenomic data

**Figure 5:**
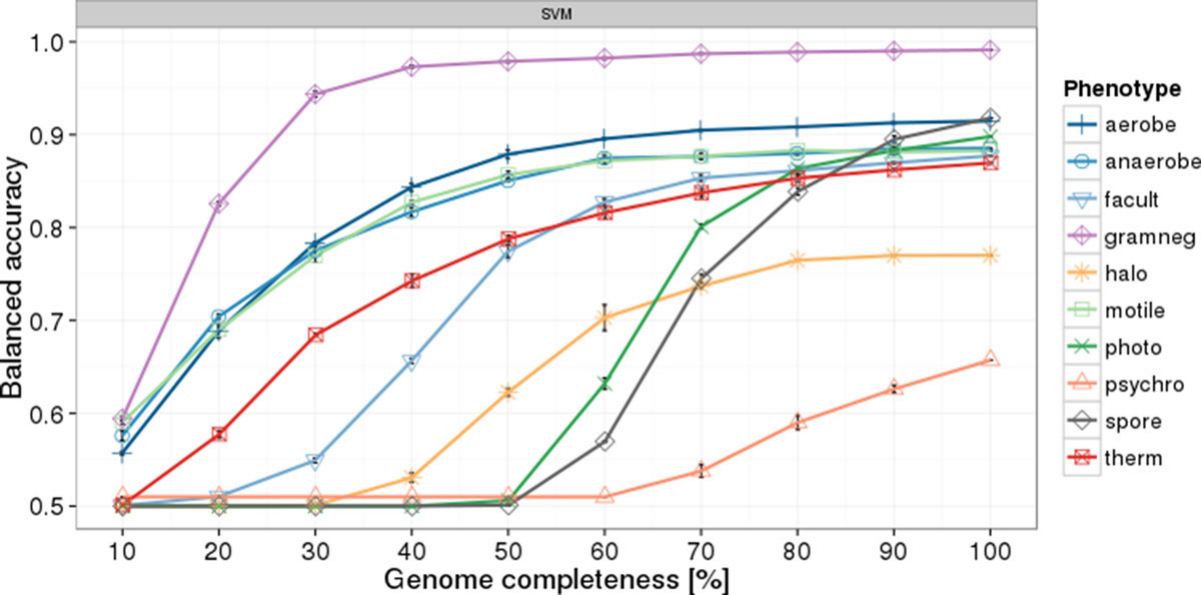
**Phenotype prediction performance for incomplete genomes**. Each point represents a cross-validation with training on complete genomes and testing on incomplete genomes. Incompleteness was simulated by random removal of x percent of all COGs in a genome. No values below 50% are observed as discussed in the main text. Error bars indicate standard deviation.

The accuracy of phenotype prediction in metagenomes is bounded by genome completeness. Bins extracted from metagenomes are often incomplete due to limitations and prediction errors of assembly and binning algorithms. We therefore assessed the prediction quality for incomplete genomes in the following procedure: at first, 10 replicates of 5-fold cross-validation were performed for the ten example traits based on eggNOG 4.0 genotype data. The results of these represent a performance baseline, i.e. an estimation of maximal accuracy per trait. In further cross-validations, phenotypic trait models were built from complete genomes in the training set. The genomes in the test sets were simulated for incompleteness by random removal of *x *percent of the total number of COGs per genome (for x in [10, 20, ..., 90]; 3 replicates). Consequently, phenotypic trait prediction was conducted on these incomplete genomes. All models show approximately sigmoidal response curves for increasing completeness (Figure 5). No values below 50 percent balanced accuracy were observed, because the SVMs tend to assign all genomes to one class in cases of insufficient data, which necessarily produces balanced accuracies of 0.5. Most models achieve almost maximal performance with 60-70 percent complete genomes. The model for detecting Gram-negative type cell envelopes works well in completeness regimes as low as 30-40 percent. On the other hand, predicting psychrophily is difficult even for complete genomes, which may be caused by the very small training set (17 positives). For the generally well performing models, only the endospore-forming and photosynthetic traits profit significantly from completeness higher than 70 percent. We conclude that sensible phenotypic trait predictions are possible for metagenomic bins that do not comprise complete genomes.

### Application on a novel trait based on genome reduction

So far we have investigated the phenotype prediction of traits, which can be inferred mainly from the presence of certain proteins. For instance, genes encoding particular porins are essential for the outer cell membrane of Gram-negative bacteria, or genes encoding specific regulators are characteristic for genomes of spore-forming microbes. However, phenotypes might also be determined by the absence of genes. In order to assess the possibility of predicting such traits, we investigated obligate intracellular lifestyle in bacteria. We hypothesize, that this trait should largely be predicted by absence of genes in the reduced genomes of such microbes. We trained a model for obligate intracellular against free-living species and facultative intracellular species (Additional file 1: Table S1). Only complete genomes were considered in this case to enable inference based on feature absence. We deemed a genome complete if at least 39 out of 40 universal prokaryotic marker COGs [31] were present. Cross-validation was performed to estimate the model quality. We observed a prediction accuracy of 0.997 ± 0.010, which is all positives as well as all negatives were predicted correctly in all cases, except for *Cand. Pelagibacter sp. IMCC9063*. This free-living organism with a small streamlined genome, which indeed shows some features of obligate intracellular microbes [32], was misclassified as obligate intracellular in 3 out of 10 folds.

Subsequently, we created a model from all 97 species with intracellular labels and performed feature ranking. All top 50 features are negative predictors, i.e. their absence is predictive for the obligate intracellular trait (Additional file 1: Table S2). We only find 2 positive predictors in the top 100 and 14 in the top 200 (data not shown). Hence, predicting this phenotype is indeed primarily based on gene absence.

**Figure 6:**
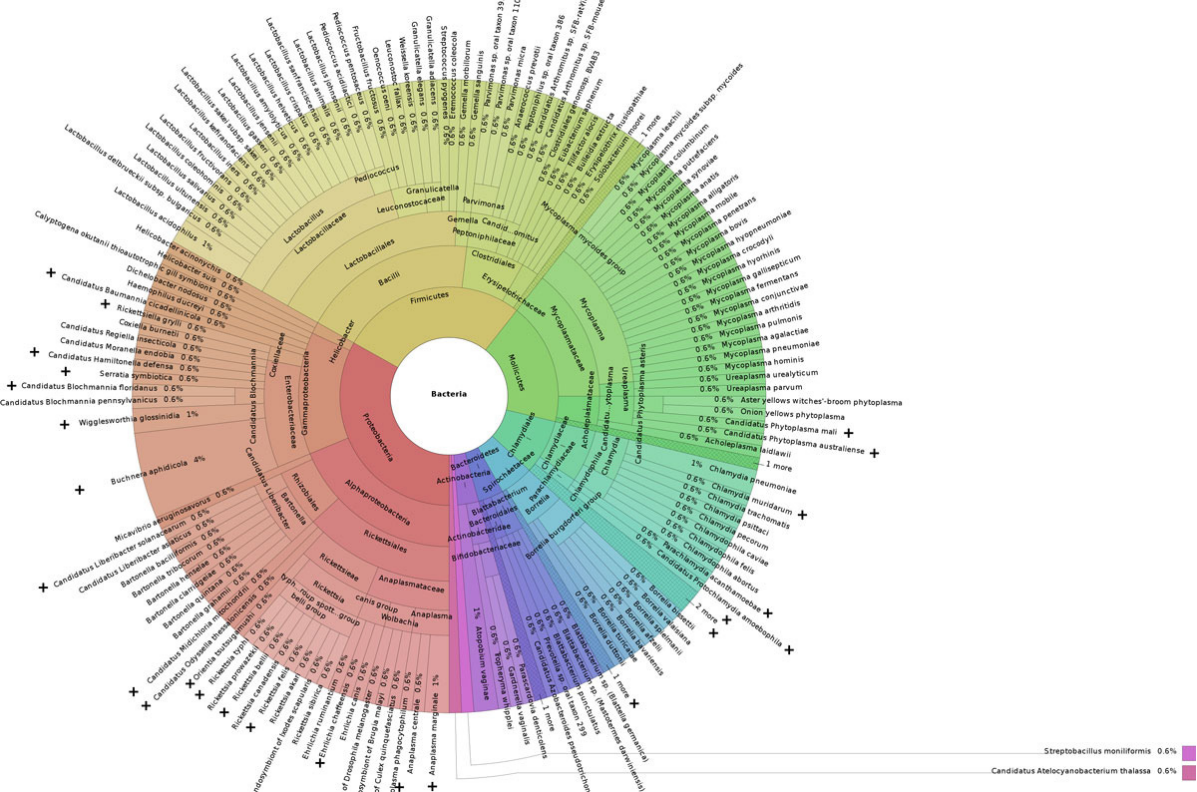
**Taxonomy of predicted obligate intracellular bacteria in eggNOG 4**.0. All species were considered whose genomes are flagged as complete based on 40 marker COGs. (+) marks indicate species also present in the obligate intracellular training set. None of the facultative intracellular or free-living species in the training set was predicted as obligate intracellular.

We tested all prokaryotic species in eggNOG 4.0 (core and periphery genomes filtered for marker COGs as described above) for obligate intracellular lifestyle. A total of 169 species is predicted intracellular, which consists of 160 bacteria (Figure 6) and 9 archaea (Additional file 1: Table S3). As expected, many known intracellular bacteria were found in our analysis, e.g. several *Mycoplasma, Chlamydia, Borrelia *and *Rickettsia *species, or *Coxiella burnetti*. Six *Bartonella *species were predicted obligate intracellular although they are truly facultative intracellular and can be cultivated in vitro. Yet they are highly fastidious and show genome features indicative of a host-integrated metabolism [33]. It is evident, that the discriminative power of the model between obligate and facultative intracellular species is suboptimal so far. This is not surprising as there is a smooth transition between these life styles, largely blurred by our ability to meet growth requirements or simulate intracellular conditions in the lab for some microbes that are host-associated in nature. In addition, the very small number of facultative intracellular organisms (six bacteria) in the training set further aggravated this distinction. A number of free-living Firmicutes, including several *Lactobacillus *species, are examples for predicted intracellular microbes, for which the reason for their unexpected classification still needs to be deciphered. A number of archaea, including Crenarchaeota from the class of Thermoprotei, have also been predicted as obligate intracellular, but in fact represent free-living microbes (Additional file 1, Table S3). This mis-classification is not surprising as metabolic pathways show pronounced differences between bacteria and archaea, with archaea lacking many classical pathways and containing unique modified variants [34]. A model based on a training set with exclusively intracellular bacteria is thus not suited for the analysis of archaeal genomes. Future models might be trained, however, for microbial eukaryotes.

## Conclusions

Inspired by the rapid progress in metagenomics, producing hundreds of high-quality genome bins from even a single modern study [e.g., [5]], we have explored how phenotypic trait prediction might better contribute to microbiology and microbial ecology. We have therefore put particular emphasis on incomplete genomes and vastly increasing data amounts. We could demonstrate the stability of the predictive power for phenotypic traits by reproducing earlier results, indicating that this method is not perturbed by the rapid growth of genome databases. A new software tool was developed that facilitates the in-depth analysis of phenotype models. It allows associating expected and unexpected protein functions with particular traits. Most of the traits can be reliably predicted in only 60-70% complete genomes, which allows reasonable predictions in genome bins from metagenomes.

We have established a new phenotypic model that predicts intracellular microorganisms. Thereby we could demonstrate that also independently evolved phenotypic traits, characterized by genome reduction, can be reliably predicted based on comparative genomics. This model is an example of a trait that cannot be associated to single functional marker genes. The predictive power of its model therefore arises from the combination of multiple (mainly absence) genotypic signals. Currently ongoing work indicates very good performance of phenotypic trait prediction also for further, ecologically important traits, as soon as sufficient training data are available. Although these models recover known functional markers, they substantially extend the marker concept by associating many further genes to the phenotypic traits. Our results suggest that the extended PICA framework developed in this study can be used to automatically annotate phenotypes in near-complete microbial genome sequences, as generated in large numbers by modern metagenomics.

## List of abbreviations used

COG: cluster of orthologous groups; NOG: non-supervised cluster of orthologous groups; LUCA: last universal common ancestor; ARM: association rule mining; CPAR: classification based on predictive association rules; SVM: support vector machine; RBF: radial basis function (Gaussian kernel).

## Competing interests

The authors declare that they have no competing interests.

## Authors' contributions

TR initiated and supervised the study. RF and TR designed the computational experiments. RF performed the computational analyses. FS and MH extracted the phenotype data for intracellular/free-living species and compiled the training dataset. RF, MH and TR analyzed the data and wrote the manuscript. All authors read and approved the final manuscript.

## Supplementary Material

Additional File 1 **Supplementary tables and supplementary figures (Microsoft Word)**. Click here for file

## Declarations

Publication costs for this article were funded by the corresponding author's institution.

This article has been published as part of *BMC Bioinformatics *Volume 16 Supplement 14, 2015: Proceedings of the 13th Annual Research in Computational Molecular Biology (RECOMB) Satellite Workshop on Comparative Genomics: Bioinformatics. The full contents of the supplement are available online at http://www.biomedcentral.com/bmcbioinformatics/supplements/16/S14.
