# Supplementary material for: Prediction of microbial phenotypes based on comparative genomics
Source: BMC Bioinformatics. 2015 Oct 2;16(Suppl 14):S1. doi: 10.1186/1471-2105-16-S14-S1 (PMC4603748; doi:10.1186/1471-2105-16-S14-S1)

**Supplementary tables**

Supplementary table 1: Training data for obligate intracellular prediction. Both free-living and facultative intracellular species are combined as 'not obligate intracellular' (negative examples).

| TaxId | Name |
| --- | --- |
| #Obligate intracellular |  |
| 37692 | Candidatus Phytoplasma mali |
| 331113 | Simkania negevensis Z |
| 1444712 | Chlamydia sp. 'Rubis' |
| 1444711 | Chlamydia sp. 'Diamant' |
| 357244 | Orientia tsutsugamushi str. Boryong |
| 36868 | Wigglesworthia glossinidia endosymbiont of Glossina morsitans |
| 673862 | Candidatus Babela massiliensis |
| 203907 | Candidatus Blochmannia floridanus |
| 91604 | Candidatus Paracaedibacter acanthamoebae |
| 1173064 | Anaplasma phagocytophilum str. JM |
| 320483 | Anaplasma marginale str. Florida |
| 452471 | Candidatus Amoebophilus asiaticus 5a2 |
| 374463 | Baumannia cicadellinicola str. Hc (Homalodisca coagulata) |
| 246273 | Wolbachia endosymbiont of Cimex lectularius |
| 186490 | Candidatus Baumannia cicadellinicola |
| 980422 | Strawberry lethal yellows phytoplasma (CPA) str. NZSb11 |
| 658172 | Candidatus Liberibacter solanacearum CLso-ZC1 |
| 97134 | Strawberry lethal yellows phytoplasma |
| 716544 | Waddlia chondrophila WSU 86-1044 |
| 696127 | Candidatus Midichloria mitochondrii IricVA |
| 985867 | Candidatus Odyssella thessalonicensis L13 |
| 572265 | Candidatus Hamiltonella defensa 5AT (Acyrthosiphon pisum) |
| 336407 | Rickettsia bellii RML369-C |
| 264201 | Candidatus Protochlamydia amoebophila UWE25 |
| 83560 | Chlamydia muridarum |
| 261317 | Buchnera aphidicola (Cinara tujafilina) |
| 1261131 | Candidatus Liberibacter americanus str. Sao Paulo |
| 1427984 | Candidatus Hepatoplasma crinochetorum Av |
| 431043 | Holospora elegans |
| 59196 | Rickettsiella grylli |
| 1274402 | Candidatus Hepatobacter penaei |
| 1478174 | Neochlamydia sp. TUME1 |
| 49893 | Holospora obtusa |
| 59748 | Candidatus Phytoplasma australiense |
| 1353976 | Neochlamydia sp. S13 |
| 257363 | Rickettsia typhi str. Wilmington |
| 205920 | Ehrlichia chaffeensis str. Arkansas |
| 568817 | Serratia symbiotica str. 'Cinara cedri' |
| 903503 | Candidatus Moranella endobia PCIT |
| 1231626 | Cardinium endosymbiont cEper1 of Encarsia pergandiella |
| 42862 | Rickettsia felis |
| 86106 | endosymbiont of Acanthamoeba sp. UWC8 |
| 765952 | Parachlamydia acanthamoebae UV-7 |
| #Facultative Intracellular |  |
| 1454642 | Salmonella enterica subsp. enterica serovar Typhimurium str. CDC 2011K-0870 |
| 661367 | Legionella longbeachae NSW150 |
| 637386 | Yersinia pestis Z176003 |
| 272624 | Legionella pneumophila subsp. pneumophila str. Philadelphia 1 |
| 243160 | Burkholderia mallei ATCC 23344 |
| 676032 | Francisella cf. tularensis subsp. novicida 3523 |
| #Free living |  |
| 240292 | Anabaena variabilis ATCC 29413 |
| 598659 | Nautilia profundicola AmH |
| 246197 | Myxococcus xanthus DK 1622 |
| 756272 | Rubinisphaera brasiliensis DSM 5305 |
| 608538 | Hydrogenobacter thermophilus TK-6 |
| 580340 | Thermovirga lienii DSM 17291 |
| 204536 | Sulfurihydrogenibium azorense Az-Fu1 |
| 644282 | Desulfarculus baarsii DSM 2075 |
| 575540 | Isosphaera pallida ATCC 43644 |
| 1144275 | Corallococcus coralloides DSM 2259 |
| 266117 | Rubrobacter xylanophilus DSM 9941 |
| 349741 | Akkermansia muciniphila ATCC BAA-835 |
| 926566 | Terriglobus roseus DSM 18391 |
| 204669 | Candidatus Koribacter versatilis Ellin345 |
| 234267 | Candidatus Solibacter usitatus Ellin6076 |
| 1520 | Clostridium beijerinckii |
| 224324 | Aquifex aeolicus VF5 |
| 316435 | Escherichia coli Nissle 1917 |
| 641491 | Desulfovibrio desulfuricans ND132 |
| 1282358 | Shigella flexneri Shi06HN006 |
| 525903 | Thermanaerovibrio acidaminovorans DSM 6589 |
| 240015 | Acidobacterium capsulatum ATCC 51196 |
| 525909 | Acidimicrobium ferrooxidans DSM 10331 |
| 452637 | Opitutus terrae PB90-1 |
| 530564 | Pirellula staleyi DSM 6068 |
| 269798 | Cytophaga hutchinsonii ATCC 33406 |
| 645127 | Corynebacterium kroppenstedtii DSM 44385 |
| 583355 | Coraliomargarita akajimensis DSM 45221 |
| 290315 | Chlorobium limicola DSM 245 |
| 156889 | Magnetococcus marinus MC-1 |
| 572480 | Arcobacter nitrofigilis DSM 7299 |
| 335992 | Candidatus Pelagibacter ubique HTCC1062 |
| 75906 | Thermocrinis ruber |
| 693979 | Bacteroides helcogenes P 36-108 |
| 123214 | Persephonella marina EX-H1 |
| 749222 | Nitratifractor salsuginis DSM 16511 |
| 682795 | Granulicella mallensis MP5ACTX8 |
| 1092 | Chlorobium limicola |
| 545695 | Treponema azotonutricium ZAS-9 |
| 638303 | Thermocrinis albus DSM 14484 |
| 324925 | Pelodictyon phaeoclathratiforme BU-1 |
| 768670 | Calditerrivibrio nitroreducens DSM 19672 |
| 579138 | Zymomonas mobilis subsp. pomaceae ATCC 29192 |
| 1002672 | Candidatus Pelagibacter sp. IMCC9063 |
| 522772 | Denitrovibrio acetiphilus DSM 12809 |
| 525898 | Sulfurospirillum deleyianum DSM 6946 |
| 289376 | Thermodesulfovibrio yellowstonii DSM 11347 |
| 315749 | Bacillus cytotoxicus NVH 391-98 |

Supplementary table 2: Top 50 most predictive features for the obligate intracellular trait as obtained from the feature-ranking algorithm.

| Rank | Group_ID | Score | Class | Group_description |
| --- | --- | --- | --- | --- |
| 1 | COG0129 | -0,01250 | NO | Dihydroxy-acid dehydratase |
| 2 | COG0069 | -0,01247 | NO | glutamate synthase |
| 3 | COG0077 | -0,01235 | NO | Prephenate dehydratase |
| 4 | COG0133 | -0,01219 | NO | The beta subunit is responsible for the synthesis of L- tryptophan from indole and L-serine (By similarity) |
| 5 | COG0065 | -0,01197 | NO | Catalyzes the isomerization between 2-isopropylmalate and 3-isopropylmalate, via the formation of 2-isopropylmaleate (By similarity) |
| 6 | COG0066 | -0,01197 | NO | Catalyzes the isomerization between 2-isopropylmalate and 3-isopropylmalate, via the formation of 2-isopropylmaleate (By similarity) |
| 7 | COG0134 | -0,01186 | NO | indole-3-glycerol phosphate synthase |
| 8 | COG0547 | -0,01186 | NO | Anthranilate phosphoribosyltransferase |
| 9 | COG0159 | -0,01186 | NO | The alpha subunit is responsible for the aldol cleavage of indoleglycerol phosphate to indole and glyceraldehyde 3- phosphate (By similarity) |
| 10 | COG0119 | -0,01183 | NO | Catalyzes the condensation of the acetyl group of acetyl-CoA with 3-methyl-2-oxobutanoate (2-oxoisovalerate) to form 3-carboxy-3-hydroxy-4-methylpentanoate (2-isopropylmalate) (By similarity) |
| 11 | COG0028 | -0,01120 | NO | acetolactate synthase |
| 12 | COG0440 | -0,01101 | NO | Acetolactate synthase small subunit |
| 13 | COG0059 | -0,01089 | NO | Alpha-keto-beta-hydroxylacyl reductoisomerase |
| 14 | COG0287 | -0,01073 | NO | prephenate dehydrogenase |
| 15 | COG0179 | -0,01071 | NO | Fumarylacetoacetate hydrolase |
| 16 | COG1052 | -0,01060 | NO | Dehydrogenase |
| 17 | COG0040 | -0,01059 | NO | Catalyzes the condensation of ATP and 5-phosphoribose 1- diphosphate to form N'-(5'-phosphoribosyl)-ATP (PR-ATP). Has a crucial role in the pathway because the rate of histidine biosynthesis seems to be controlled primarily by regulation of HisG enzymatic activity (By similarity) |
| 18 | COG0107 | -0,01059 | NO | IGPS catalyzes the conversion of PRFAR and glutamine to IGP, AICAR and glutamate. The HisF subunit catalyzes the cyclization activity that produces IGP and AICAR from PRFAR using the ammonia provided by the HisH subunit (By similarity) |
| 19 | COG0106 | -0,01059 | NO | phosphoribosylformimino-5-aminoimidazole carboxamide ribotide isomerase |
| 20 | COG0131 | -0,01059 | NO | imidazoleglycerolphosphate dehydratase |
| 21 | COG0141 | -0,01059 | NO | Catalyzes the sequential NAD-dependent oxidations of L- histidinol to L-histidinaldehyde and then to L-histidine (By similarity) |
| 22 | COG0118 | -0,01059 | NO | IGPS catalyzes the conversion of PRFAR and glutamine to IGP, AICAR and glutamate. The hisH subunit provides the glutamine amidotransferase activity that produces the ammonia necessary to hisF for the synthesis of IGP and AICAR (By similarity) |
| 23 | COG0137 | -0,01033 | NO | Citrulline--aspartate ligase |
| 24 | COG0067 | -0,01027 | NO | glutamate synthase |
| 25 | COG0070 | -0,01027 | NO | glutamate synthase |
| 26 | COG0111 | -0,01024 | NO | Dehydrogenase |
| 27 | COG2022 | -0,00992 | NO | Catalyzes the rearrangement of 1-deoxy-D-xylulose 5- phosphate (DXP) to produce the thiazole phosphate moiety of thiamine. Sulfur is provided by the thiocarboxylate moiety of the carrier protein ThiS. In vitro, sulfur can be provided by H(2)S (By similarity) |
| 28 | COG0714 | -0,00991 | NO | ATPase associated with various cellular activities |
| 29 | COG0139 | -0,00981 | NO | Phosphoribosyl-amp cyclohydrolase |
| 30 | COG0640 | -0,00972 | NO | Transcriptional regulator, arsr family |
| 31 | COG0135 | -0,00964 | NO | N-(5'-phosphoribosyl)anthranilate isomerase |
| 32 | COG0352 | -0,00948 | NO | Condenses 4-methyl-5-(beta-hydroxyethyl)thiazole monophosphate (THZ-P) and 2-methyl-4-amino-5-hydroxymethyl pyrimidine pyrophosphate (HMP-PP) to form thiamine monophosphate (TMP) (By similarity) |
| 33 | COG0299 | -0,00941 | NO | phosphoribosylglycinamide formyltransferase |
| 34 | COG2252 | -0,00938 | NO | Xanthine uracil vitamin C permease |
| 35 | COG0038 | -0,00937 | NO | chloride channel |
| 36 | COG0041 | -0,00931 | NO | Catalyzes the conversion of N5-carboxyaminoimidazole ribonucleotide (N5-CAIR) to 4-carboxy-5-aminoimidazole ribonucleotide (CAIR) (By similarity) |
| 37 | COG0263 | -0,00930 | NO | Catalyzes the transfer of a phosphate group to glutamate to form glutamate 5-phosphate which rapidly cyclizes to 5- oxoproline (By similarity) |
| 38 | COG0014 | -0,00930 | NO | Catalyzes the NADPH dependent reduction of L-gamma- glutamyl 5-phosphate into L-glutamate 5-semialdehyde and phosphate. The product spontaneously undergoes cyclization to form 1-pyrroline-5-carboxylate (By similarity) |
| 39 | COG0047 | -0,00926 | NO | phosphoribosylformylglycinamidine synthase |
| 40 | COG0034 | -0,00926 | NO | glutamine phosphoribosylpyrophosphate amidotransferase |
| 41 | COG0046 | -0,00926 | NO | phosphoribosylformylglycinamidine synthase |
| 42 | COG0150 | -0,00926 | NO | phosphoribosylaminoimidazole synthetase |
| 43 | COG0151 | -0,00926 | NO | Phosphoribosylglycinamide synthetase |
| 44 | COG0476 | -0,00920 | NO | UBA THIF-type NAD FAD binding protein |
| 45 | COG0414 | -0,00916 | NO | Catalyzes the condensation of pantoate with beta-alanine in an ATP-dependent reaction via a pantoyl-adenylate intermediate (By similarity) |
| 46 | COG0413 | -0,00916 | NO | Catalyzes the reversible reaction in which hydroxymethyl group from 5,10-methylenetetrahydrofolate is tranferred onto alpha-ketoisovalerate to form ketopantoate (By similarity) |
| 47 | NOG00108 | -0,00915 | NO | Dehydrogenase |
| 48 | COG0347 | -0,00910 | NO | Nitrogen regulatory protein pii |
| 49 | COG0031 | -0,00908 | NO | cysteine synthase |
| 50 | COG0351 | -0,00906 | NO | phosphomethylpyrimidine kinase |

Supplementary table 3: Predicted obligate intracellular archaea in eggNOG 4.0.

| TaxId | Name |
| --- | --- |
| 84599 | Staphylothermus hellenicus |
| 2280 | Staphylothermus marinus |
| 477693 | Desulfurococcus kamchatkensis |
| 2275 | Desulfurococcus mucosus |
| 54254 | Thermosphaera aggregans |
| 54248 | Hyperthermus butylicus |
| 2269 | Thermofilum pendens |
| 242703 | Acidilobus saccharovorans |
| 274854 | uncultured marine group II euryarchaeote |

**Supplementary figures**

Supplementary figure 1: Obligate intracellular species in the training set (total number: 43)


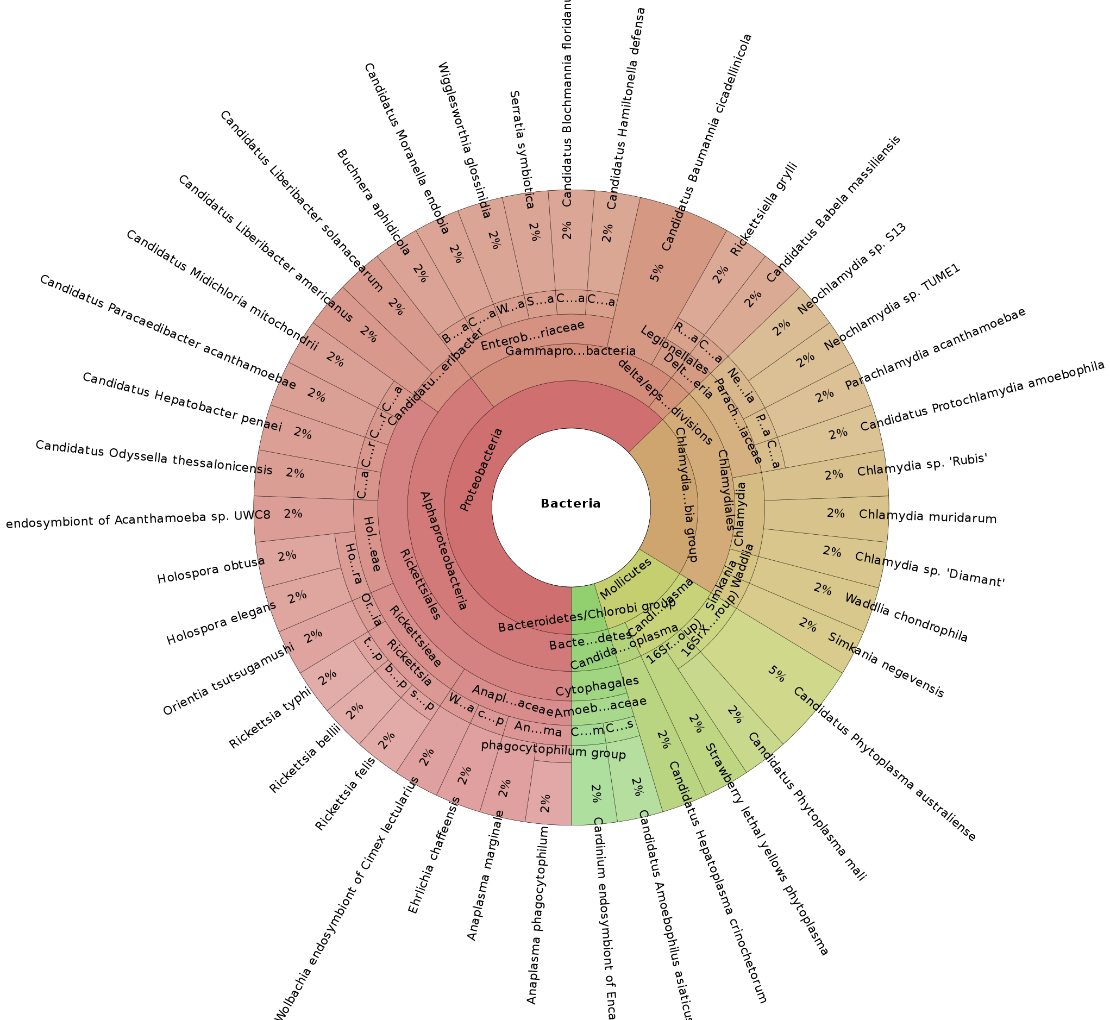


Supplementary figure 2: Facultative intracellular species in the training set (total number: 6).
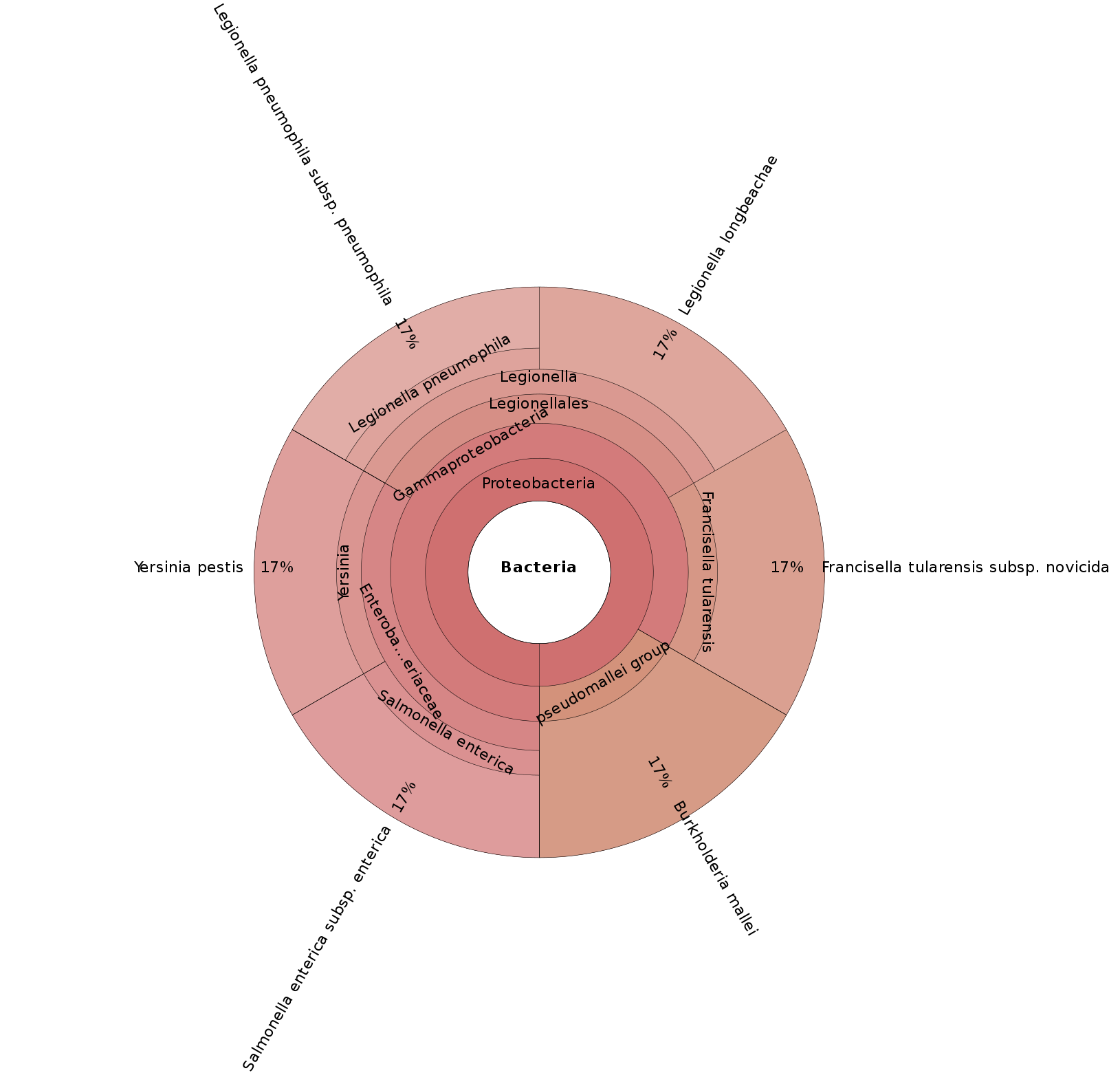


Supplementary figure 3: Free living species in the training set (total number: 48).


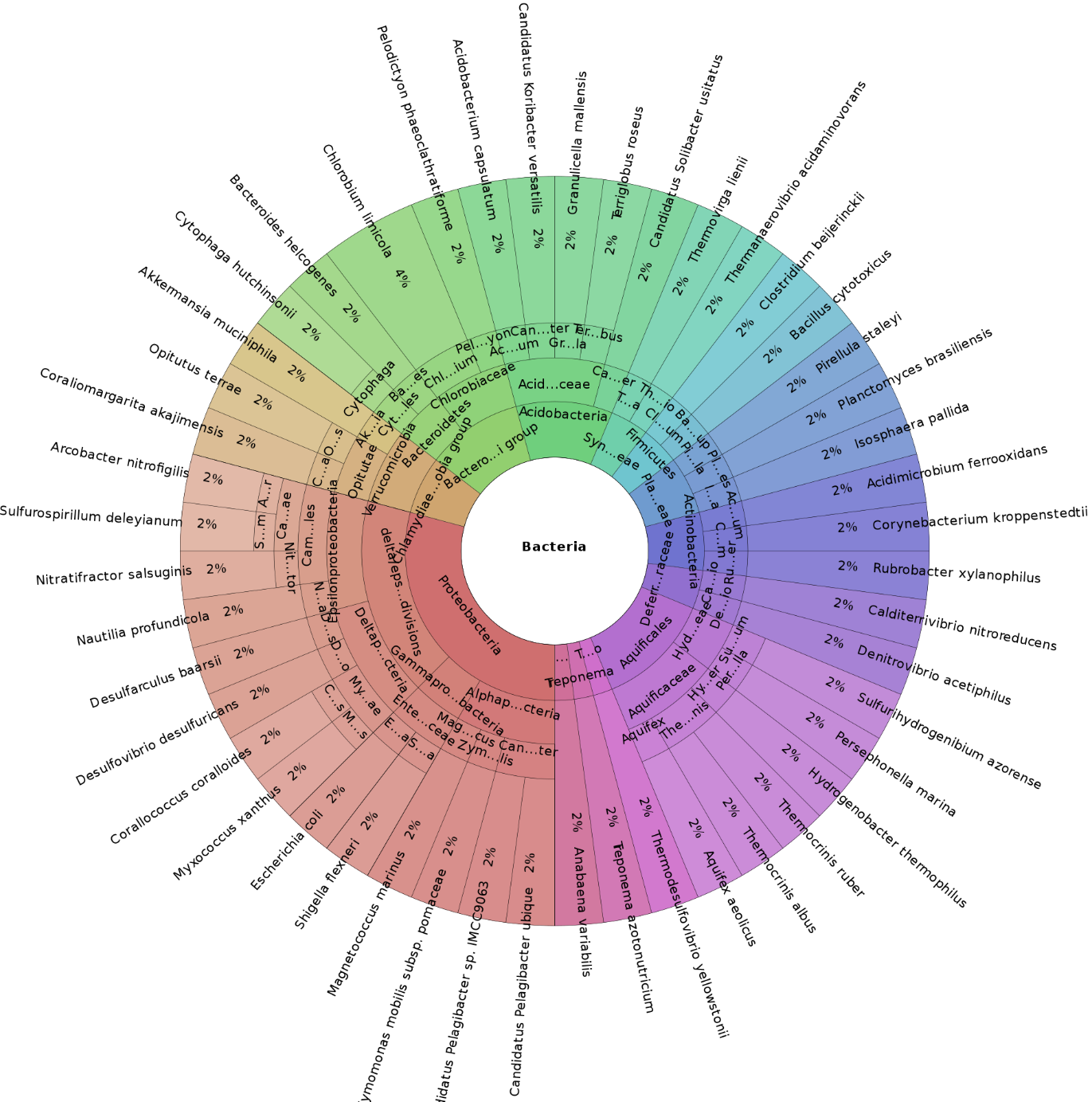

Supplement: Additional File 1 — Supplementary tables and supplementary figures (Microsoft Word). [file 1471-2105-16-S14-S1-S1.docx]
